# Levels of Airborne Soybean Allergen (Gly m 1) in a Brazilian Soybean Production City: A Pilot Study

**DOI:** 10.3390/ijerph17155381

**Published:** 2020-07-26

**Authors:** Cinthya Covessi Thom de Souza, Nelson Augusto Rosário Filho, Juliana Francis de Camargo, Ricardo Henrique Moreton Godoi

**Affiliations:** 1 Department of Pediatrics, Federal University of Paraná, Curitiba 80060-240, Brazil; 2 Departament of Environmental Engineering, Federal University of Paraná, Curitiba 80060-240, Brazil; julianafcamargo01@gmail.com (J.F.d.C.); rhmgodoi@gmail.com (R.H.M.G.)

**Keywords:** allergy, soybean hulls, Gly m 1, monitoring, air samples

## Abstract

Asthma epidemics have been shown to be related to where soybeans are loaded and handled, but data are scarce in the literature. This pilot study evaluated the levels of Gly m 1 in dust samples collected in Maringá, Brazil, a city with high soy production and processing. A dust impactor was used to collect seven isolated samples during 2015 and 2016. Samples were analyzed by an ELISA (enzyme-linked immunosorbent assay) detection method. Gly m 1 was found in all samples, ranging from 0.82–24.38 ng/m^3^ (median 2.41), regardless of the month or year evaluated. The levels of Gly m 1 were considered low, but the concentrations required to cause sensitization and symptoms are uncertain.

## 1. Introduction

Soybean (Glycine max) is the principal oleaginous species currently cultivated, and Brazil is the world’s second largest soybean producer. Nevertheless, soybean is composed of a range of proteins with a high allergenic potential [1].

There are three distinct groups of individuals affected by soybean protein allergens. The first comprises young infants with early gastrointestinal sensitization to stable soybean allergens (Gly m 5, 6, 8) who present food allergies with severe systemic reactions. The second comprises atopic individuals sensitized to birch pollen (Bet v 1) with cross-reactivity to soybean PR-10, Gly m 4. These individuals show oropharyngeal and sometimes systemic allergic symptoms. The last group shows respiratory symptoms to soybean dust. Exposure to soybeans induces IgE-mediated sensitization to hull allergens (Gly m 1, Gly m 2), especially in occupationally exposed individuals [2].

The first report of asthma caused by exposure to soybean dust was described in 1934 [3] in five workers exposed to soybean flour. During the 1980s, some asthma outbreaks were associated with the inhalation of soybean dust during soy unloading in some European harbors, especially in Barcelona, where 26 outbreaks of asthma affected a total of 688 individuals [4,5]. Thus, occupational asthma and rhinitis due to soybean dust proteins have been considered in predisposed individuals, although the concentrations of soybean hull allergens that can cause sensitization and symptoms are still unknown.

In the late 1980s, Rodrigo et al. identified patients who suffered from soy epidemic asthma, which produced specific IgEs to low-molecular-mass proteins in soybean hulls [6]. In 1992, Gonzalez et al. identified Gly m 1 as the main allergen responsible for these asthma outbreaks [7,8]. Soybean IgE antibodies were identified in 84.9% of epidemic asthmatics population in Barcelona by radioimmunoassay, compared with 5.8% of non-epidemic asthmatic population. In 1995, this low-molecular-weight allergen with approximately 8 kDa, Gly m 1, was recognized as a producer of specific IgE in 90.5% of epidemic asthmatics by ELISA and was shown to be different from those causing soy food allergies [9]. Then, in 2000, Gonzalez et al. developed a monoclonal antibody-based method to quantify Gly m 1 with high sensitivity [10].

Despite the importance of this crop in Brazil, it remains unknown whether Gly m 1 is present in the air in regions where soybeans are widely cultivated, harvested and handled. Previous Brazilian research found greater sensitization among people living near areas of soybean plantation, although the concentrations of airborne allergens have not been evaluated [11].

Maringá is a Brazilian grain-producing city with about 400 thousand inhabitants. A large part of its agribusiness regions is located next to the main areas of the products and direct purchases from the producers. Thus, the production and logistic chain involved ends up concentrating cooperatives and processing industries close to or even within urban areas. It is estimated that a population of about 100,000 inhabitants lives within a 5 km radius of the main cooperative in the city,. In addition, highways very close to the city transport soy to industries inside and outside the city. The present pilot study was undertaken to measure Gly m 1 in the atmosphere of Maringá by a monoclonal antibody-based method.

## 2. Materials and Methods

A method based on air sampling with ELISA-based detection was used to measure Gly m 1. Teflon filters (Pallflex^®^ membrane filters) were used, and air samples were obtained using an impactor of total particulate that was located outside, 60 cm from the ground, with a flow rate of 28 L/min. The collection point was located in an urban region of Maringá, latitude 23.41° S, longitude 51.97° W, 521 m above sea level and 2.5 km from the most important soybean industry in the region. The samples were collected once a month for 24 h from September 2015 to April 2016, totaling seven samples. After collection, filters were stored and frozen (−20 °C) until analysis. For preparing the ELISA, small pieces (3 cm^2^) of the filters were incubated with 3 mL of PBS (phosphate buffered saline), containing 1% BSA (bovine serum albumin) and 0.1% Tween 20 (PBS-BSA-T), in an orbital shaker overnight at 4 °C. Afterward, the solution was recovered and filtered through a 0.22-mm filter. Then, the samples were analyzed by ELISA for Gly m 1. It was a direct ELISA with a quantification limit of 0.4 ng/mL. This method is highly specific for Gly m 1 and does not lead to cross-reactivity or false positives with other allergens. The test was performed with ELISA plates pre-sensitized with a monoclonal antibody, anti-Gly m 1. The wells then were washed and sequentially incubated in duplicate with samples and references (standard, controls and blank filters), and biotin-labeled with anti-Gly m 1 mAb 1G10 and streptavidin/peroxidase conjugate. Finally, the wells were incubated for 30 min at room temperature in the dark with a solution of o-phenylendiamine, and the color reaction was stopped by adding HCl. The optical density was then read at 490 nm with a 650-nm reference filter (previously described in Gonzalez 2000) [10].

## 3. Results

Gly m 1, ranging from 0.82–24.38 ng/m^3^, with a median of 2.41 ng/m^3^, was found in all seven samples. The highest concentration was found in September 2015, and the lowest in March 2016 (Table 1).

## 4. Discussion

The results of this study confirm, for the first time, the presence of Gly m 1 in the atmosphere in Brazil. Although Brazil, and in particular the city of Maringá and its surroundings, is a major producer of soybeans, thus far, there have been no qualitative or quantitative assessments of soybean dust allergens in Brazil.

The ELISA technique used to evaluate the presence of Gly m 1 is highly sensitive, which allowed us to identify this allergen in all samples, even in very small quantities and in different months, regardless of the climatic conditions.

Theoretically, in the months of soybean harvest (February, March and April), there would be a greater propensity for the dispersion of soy allergens in the air. However, we did not find differences between these months and the others, except for the September sample, in which the concentration was higher than those for the other months. Nonetheless, the greater dispersion of allergens during the harvest period should not be easily demonstrated, because soybeans are stored, handled and processed throughout the year. Presumably, this highest concentration found in September was due to the climate, humidity and winds. It was not possible to establish statistical correlations between the climatic conditions and samples because there were too few samples.

The concentration of Gly m 1 that causes symptoms has not yet been established. Gijzen et al. [12] found a concentration of 73 ng/m^3^ in Canada during harvest. Antonicelli et al. [13] found concentrations higher than 100 ng/m^3^ in the port of Ancona, Italy. Neither of these values correlated with asthma outbreaks. This is possibly due to the environmental factors (climate, humidity, pollution, wind direction) inherent in each site, which may or may not lead to the dispersion of allergens as well as the triggering of symptoms.

Considering the number of patients who presented asthma during the outbreak in Barcelona, Spain, the frequency of soybean dust allergenicity has been estimated to be approximately 1 per 1700 people [14]. Current knowledge is not clear regarding how much Gly m 1 is needed to cause allergic sensitization.

Codina et al. [15] (2000) found that sensitization was present in Argentinians with asthma who had indirect exposure to soybean dust (20.3% with a positive prick test) and even in urban individuals who were not exposed to soybean dust (8.4% with a positive prick test). They concluded that there was a high prevalence of sensitivity to soybean hulls in subjects with asthma or allergic rhinitis, and an association between sensitivity to soybean hulls, the severity of asthma and the level of exposure to soybean dust. In Brazil, Pinto et al. [11] (2007) showed that soybean workers, as well as the surrounding population, from rural or urban areas had 15% and 22% soybean sensitizations. Despite the low concentrations of Gly m 1 found in this study, it is possible that there is a high level of sensitization to the Gly m 1 soybean hull allergen in the Maringá population. As the concentrations found were not enough to cause symptoms, there is a potential for clinical investigation in situations of increasing concentrations as to when this sensitized population may experience symptoms.

Furthermore, sensitization and symptoms are not exclusively related to the concentration of airborne Gly m 1. In 1998, Codina et al. [16] showed that soybean hull allergenicity is affected by heat and suggested that warming during the storage and transport of soybeans could produce two new allergen determinants or increase the epitope exposure by conformational changes. In addition, genetic predisposition could contribute to the response of some patients with asthma to exposure to soybean dust. Soriano et al. [5] found that the risk of epidemic asthma was mainly associated with HLA (human leukocyte antigen) DRB1*13.

The limitation of this study is the small number of samples. Nonetheless, the data were sufficient to detect the environmental presence of Gly m 1.

## 5. Conclusions

In conclusion, these results showed the constant presence of Gly m 1, suggesting that this allergen could be responsible for the allergic sensitization of susceptible individuals living in Maringá. However, the clinical impact of this exposure was not assessed in this study. Other studies with frequent measurements and climatic correlation as well as data on the sensitization of this population are needed to improve understanding of the real impact of this allergen in this population.

## Figures and Tables

**Table 1:** Concentration of Gly m 1 in each monthly sample.

Sample Filter	Gly m 1 (ng/m^3^)	Month
Blank (control)	0.13	---
1	24.38	Sept 2015
2	3.95	Oct 2015
3	2.13	Dec 2015
4	2.41	Jan 2016
5	1.27	Feb 2016
6	0.82	Mar 2016
7	5.88	Apr 2016
